# Spontaneous Epidural and Corpus Callosum Hemorrhage in Sickle Cell Disease – An Unusual Presentation in a Ghanaian Patient

**DOI:** 10.7759/cureus.12292

**Published:** 2020-12-26

**Authors:** Solomon N Kotey, Nkechi O Dike, Edem Nani, Kwaku Nyame

**Affiliations:** 1 Emergency Department, Nyaho Medical Center, Accra, GHA; 2 School of Medical Sciences, University of Cape Coast, Cape Coast, GHA; 3 Emergency Department, Greater Accra Regional Hospital, Accra, GHA; 4 Emergency Department, Komfo Anokye Teaching Hospital, Kumasi, GHA

**Keywords:** vaso-occlusive crisis, sickle cell disease, sickle cell disease complications, spontaneous intra-cranial hemorrhage, corpus callosum hemorrhage, epidural hemorrhage

## Abstract

Spontaneous intracranial bleed in sickle cell disease is a rare presentation and complication of the disease, with a few cases presenting with epidural hematoma. We present an 18-year-old boy with sickle cell, hemoglobin FS, who presented with non-traumatic scalp swelling, headaches, and vomiting six days following an episode of vaso-occlusive crisis with bone pain. A head CT scan showed extensive epidural hematoma with mass effect and acute corpus callosum bleed. The patient, however, had a cardiac arrest with unsuccessful resuscitation before neurosurgical interventions could be instituted. Of all reported cases, none has reported associated bleeding in the corpus callosum, making our case the firstwith such a combination, possibly worsening the outcome.

## Introduction

Sickle cell disease (SCD) is a hemoglobinopathy that results from the substitution of valine for glutamic acid at position 6 of the beta-globin chain with the formation of sickle hemoglobin (HbS). It is more common in Africans and people of Mediterranean descent. Sickle cell hemoglobin is an unstable moiety and red blood cells (RBCs) that have high amounts of HbS deform into a sickle shape in low oxygen states [1].

The change in the structure of the hemoglobin results in sickle cell vaso-occlusive crisis and other acute and long-term complications. Strokes, including hemorrhagic, have long been identified as a cerebrovascular complication of sickle cell disease [2]. Fetal hemoglobin (hemoglobin F) is thought to be protective in sickle cell patients, with hemoglobin FS (HbSF) patients known to experience fewer and milder complications than HbSS and HbSC [3]. Commonly documented cerebrovascular complications of SCD are ischemic strokes (75%), intra-parenchymal hemorrhage (25%), and more rarely sub-arachnoid hemorrhage, sub-galeal hematoma, and extra-dural/epidural hematoma [4].

We present a case of spontaneous epidural hematoma with associated corpus callosum hemorrhage in a sickle cell disease patient with genotype FS, seen at the Emergency Department of Komfo Anokye Teaching Hospital, Kumasi. This is a rare presentation and complication of sickle cell in Ghana, and the first documentation of such a case report.

## Case presentation

An 18-year-old male with sickle cell genotype FS was referred to the Emergency Department after he was managed for vaso-occlusive crises on intravenous fluids, analgesia, antibiotics, and antimalarial, and the patient's condition was not improving. He had developed bone pain in both upper and lower limbs six days prior to being referred to our facility. He had earlier been seen as an outpatient, however, he presented back to the private facility with increasingly severe joint aches, and admitted for four days. Before referral, whilst on admission, the patient was noted to have developed non-traumatic scalp swellings. He also complained of headaches and vomiting. The vomitus was non-bloody and non-bilious. The patient was initially conscious then developed a reduced level of consciousness and was thus referred to our facility.

On presentation, the patient was tachycardic with a heart rate of 140 bpm, conjunctival icterus, normotensive, and febrile. There were five separate swellings with an average size of 3cm on the head; four were frontal and one was left parieto-occipital. Three of the swellings had soft consistency and the other two had bony-like consistency. The Glasgow Coma Score was 10 (M5V1E4). Both pupils were 3mm and reactive to light. There was a grade one pansystolic murmur loudest at the mitral area. Other examination findings were not significant. The patient was put on antibiotics to cover central nervous system infection, intravenous fluids, and analgesia. The initial working diagnosis was sickle cell anemia with vaso-occlusive and hemolytic crises; and altered mental state with differentials of meningitis, cerebral abscess, and cerebrovascular accident (CVA). A contrast CT scan was requested. His hemoglobin level (Hb) was 6.2g/dl and he was hemo-transfused 2 units of whole blood.

**Figure 1:**
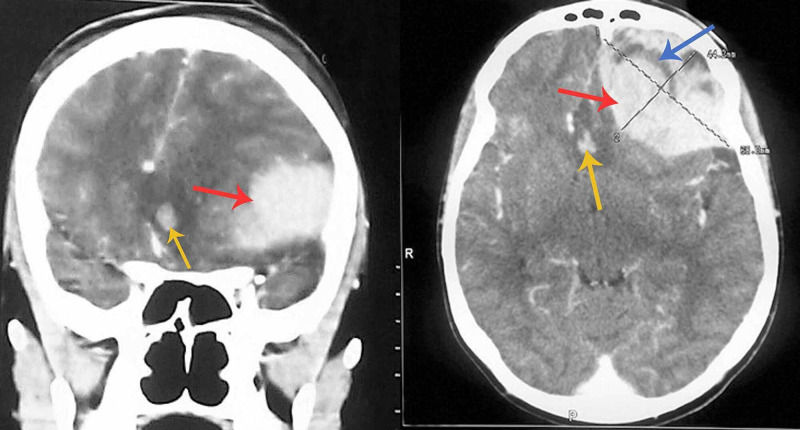
Patient's head CT scan showing left frontal epidural bleed (red arrow) and the swirl sign (blue arrow) with mass effect; and corpus callosal bleed (yellow arrow). (Left image: coronal view. Right image: axial view) CT - Computerized Tomography

His CT scan, currently not covered by the national health insurance scheme, was delayed and done on the second day of admission. It showed acute hemorrhage in the corpus callosum, extensive acute left frontal epidural hemorrhage with severe mass effect, and swirl sign suggestive of ongoing bleeding (Figure 1). There was generalized sulcal effacement suggestive of cerebral edema. The bone window showed sub-galeal hematoma. There was no clear evidence of skull bone infarction.

The patient was started on 10% mannitol IV 1g/kg body weight pending surgical evacuation of the epidural hematoma. Neurosurgical intervention was deferred due to concerns about the patient’s clotting profile. Due to unavailability in the facility, the test had to be sent to an external laboratory to run and was not immediately accessible to the neurosurgeons.

The patient ceased breathing a few hours after obtaining the CT scan reports and went into a cardiac arrest with asystole rhythm. Cardiopulmonary resuscitation was started following the Advanced Cardiac Life Support (ACLS) algorithm but was unsuccessful at restoring spontaneous circulation. The family elected against an autopsy, hence a post-mortem examination was not done.

## Discussion

Spontaneous epidural hematoma in sickle cell disease is a rare complication [4]. Different mechanisms have been postulated for the development of epidural hematomas in SCD. One of the postulates describes skull bone infarction with cortical disruption and periosteal elevation. Another postulate describes the disruption of the skull cortex by hemopoietic proliferation and resultant bleeding into the epidural space.

Skull infarction 

The majority of the previous reports identify skull bone infarction in the same area as the extradural hematoma and the commonest interpretation is that extradural hematomas are a complication of bone infarction disrupting the cortical bone, causing periosteal elevation and subsequent bleeding into the extradural space. There could also be a spontaneous rupture of epidural vessels in the vicinity of the infarcted bone [5-9].

Hemopoietic tissue proliferation

This suggests that patients with SCD have abnormal skull anatomy due to chronic medullary hematopoiesis. In response to acute anemia, there is rapid hemopoietic tissue proliferation and expansion, which could disrupt the skull cortex and precipitate extravasation of blood and marrow into the sub-galeal and epidural spaces [10,11].

Presentation

Patients typically present with vaso-occlusive crises, which are not amenable to their regular analgesics; and may experience a rapid decline in the neurologic state (as per the Glasgow Coma Scale score) after complaints of headaches [12-15], as seen with our patient. The characteristic lucid interval in extradural hemorrhage is absent. They may also present with hemiparesis or hemiplegia.

Some patients present with sub-galeal hematomas which appear as spontaneously-arising soft scalp swellings that develop over a short period of time. The presence of these scalp swellings may be evidence of sub-galeal bleeds or underlying bone infarction. Sub-galeal hematomas are more associated with epidural hematomas with underlying bone infarction. Mishra et al. [9] analyzed prior published reports and found skull bone infarction in seven out of nine cases reviewed. From the reviewed case reports in adults, the mean age at presentation was found to be 19 years, which is comparable to our patient’s reported age of 18 years.

Other indirect pointers include a rapid drop in Hb and Hemoglobin which cannot be solely attributed to vaso-occlusive crisis in a sickle cell patient with neurologic symptoms; and proptosis. New proptosis with neurologic symptoms in a sickle cell patient might signify retrobulbar hematoma associated with extradural hemorrhage [7,16]

Important labs to request include full blood count (FBC), liver function tests (LFTs), clotting profile, and lactic acid dehydrogenase (LDH). Ultrasound can be useful in determining if scalp swellings are hematomas [17]. CT scans are the definitive diagnostic modality.

Our patient was initially being managed as having meningitis which is a more common presentation seen in Ghana. Extradural hemorrhage is rare and requires a high index of suspicion. Of the published cases of sickle cell patients with spontaneous epidural hematomas, none so far has reported associated bleeding in the corpus callosum, making our case the first with such a combination, possibly worsening the outcome.

Management

Craniotomy and surgical evacuation is the definitive treatment for SCD patients with epidural hematoma who are unconscious. In cases that underwent surgical evacuation, full recovery has been documented [12,14]. Oka et al. reported a case that was managed conservatively with recovery and spontaneous resolution of the hematoma [15]. Although this was not their primary treatment choice, the management option stemmed from the patient’s objection to hemo-transfusion if necessitated during surgery, for which reason the surgery could not be performed, and was given analgesics, anti-inflammatory, and vasodilator drugs. He was then followed up clinically every month with good neurologic outcome by the fourth year. It should also be noted that the patient was managed conservatively and was fully conscious all through the period he had the hematoma, indicative that the choice for conservative management is best based on the conscious level of the patient or whilst awaiting neurosurgical intervention.

## Conclusions

Spontaneous intracranial hemorrhages, including the more commonly occurring epidural hematomas, are a life-threatening complication of SCD. This is the first reported case from Ghana and the third from sub-Saharan Africa despite a higher burden of SCD. The rareness of this complication may have resulted in a lower index of suspicion in this patient. Since sub-Saharan Africa has the majority of the burden of sickle cell disease, it is necessary that physicians develop high alertness to complications of the disease. Physicians in these regions should take advantage of the increased availability of CT scans to look for these complications in patients presenting with headaches, reduced consciousness, or suspicious scalp swellings in the setting of sickle cell disease since there is evidence that early detection and diagnosis, and prompt intervention significantly improves patient outcomes.
